# Viewing Decellularized Amniotic Membrane Through the Lens of Coupled Scaffolding and Drug Delivery Systems in Regenerative Medicine

**DOI:** 10.1155/term/8818058

**Published:** 2025-07-23

**Authors:** Fatemeh Alibabaei-Omran, Nima Javanmehr, Atiyeh Al-e-Ahmad, Ebrahim Zabihi, Tohid Najafi

**Affiliations:** ^1^Cellular and Molecular Biology Research Center, Health Research Institute, Babol University of Medical Sciences, Babol, Iran; ^2^Student Research Committee, Babol University of Medical Sciences, Babol, Iran; ^3^Department of Pharmacology & Toxicology, School of Medicine, Babol University of Medical Sciences, Babol, Iran; ^4^USERN Office, Babol University of Medical Sciences, Babol, Iran; ^5^Department of Clinical Biochemistry, School of Medicine, Babol University of Medical Sciences, Babol, Iran; ^6^Laboratory and Pathology Services, UChicago Medicine Adventhealth, La Grange, Illinois, USA

**Keywords:** amniotic membrane, cell-based therapy, decellularization, drug delivery system, regenerative medicine, tissue regeneration

## Abstract

Regenerative medicine (RM) exploits stem cells to construct biological replacements and repair damaged tissues, offering an alternative to daunting organ transplantation. Even while RM has advanced quickly, building an entire organ remains beyond our capabilities. Experts are thus investigating the adoption of biologically generated composites that preserve the tissue's crucial physiological, morphological, and mechanical characteristics. Noncellular tissues like extracellular matrix offer cells a milieu similar to their physiological niche, becoming a promising substitute for synthetic composites. In this context, amnion, the membrane enclosing the fetus, is a great contender since it is widely obtainable and economical. Given its biochemical and anatomic characteristics, and the extensive supply of stem cells, growth factors, and matrix proteins, the amnion is considered a fantastic candidate to employ in RM. Decellularized amniotic membrane (DAM) has many uses as two- and three-dimensional scaffolds, anchoring for cell adhesion and expansion for tissue regeneration, and as carrier systems for cell and drug cargoes. The present research aims to assess the recent surge in DAM-RM research, potentially to get beyond the existing barriers impeding the RM's clinical translation landscape. The present paper draws a comprehensive picture of the experimental evidence and clinical trials regarding exploiting DAM in RM.

## 1. Introduction

When the body's capacity to heal and rebuild wounded tissues is restricted due to organ malfunctioning and failure, autologous and allogeneic tissue/organ transplant is the conventional intervention [1]. Nevertheless, a scarcity of appropriate donors and immense immune reactions incurring rejection and graft-versus-host disease (GVHD) continue to be significant transplant obstacles [2]. In this realm, regenerative medicine (RM) has arisen as a novel and diverse discipline that integrates cells, extracellular matrix (ECM) biomimetics, and cytokines to restore tissues' homeostasis and mend pathologies [3]. Tissue engineering (TE) exploits stem cells' (SCs') high proliferation and differentiation potencies, cultivated on synthetic scaffolds and accompanied by growth factors to create physiological replacements and enhance severed tissues' functioning [4].

An improved understanding of cell sources and alternative biomaterials might yield further options for RM progress. Over a century has passed since amniotic membrane (AM) first saw usage as a medical substance. In the 1920s and 1930s, AM was harnessed to repair burn and ulcer wounds. In the years after, AM has grown into the foundation of countless vital applications, such as biological dressing for mucocutaneous lesions (such as scalds, diabetic ulcers, and bedsores) by accelerating re-epithelization, decreasing discomfort, and lessening fibrotic scars [5]. AM has garnered attention in this realm, providing a putative bioengineered therapeutic avenue to counter the limits of the RM triad, SCs, scaffolds, and growth factors. Because the AM is conventionally regarded as a post-parturition waste, and due to its highly cost-effective availability and minimal ethical concerns, researchers denote that AM is a solid bet in the burgeoning field of RM [6]. The AM comprises an epithelium formed upon a robust basement membrane and a stroma interspersed by fibroblasts [7]. There is undoubtedly pronounced potential for AM to transform TE technology, partly due to its minimal antigenicity and distinct antiangiogenic, antimicrobial, and immune modulatory impacts to abate inflammatory responses and fibroblast activity [8]. Its rife supply of SC and cytokine and growth factors influence adjacent and remote cells to boost recuperation and re-epithelization in severed tissues [9]. Due to its concrete mechanical strength, AM is harnessed as a substantially immune-privileged biological scaffold, an unparalleled asset for cell migration and proliferation [10]. Moreover, the pronounced entrapment capacity of amnion renders it helpful in delivering nanoparticle (NP) and drug cargoes [11]. Research on the application of AM scaffolding on corneal reconstruction draws a consistent picture with these sentiments, AM could efficiently be embedded with limbal stem cells (LSCs) in vitro and grafted on the eye with promising outcomes [12].

On the other hand, AM's share of the RM market is in jeopardy due to its rather challenging storage requirements, such as −80°C freezers and sterile vials, contributing to its rising costs [13]. Decellularized amniotic membrane (DAM), separated from AM by eliminating most cell constituents, is less immunogenic than AM [14]. Furthermore, AM is shown to be anticarcinogenic and antibacterial, with immunomodulatory effects, resulting in minimal moral concerns [15].

Decellularization is a critical process in preparing biological tissues for use as scaffolds in RM [16]. The aim is to remove all cellular components while preserving the ECM structure and function [17].

Employing DAM on its own or coated with other kinds of cells and biosynthesized cargoes has yielded promising results useful in biomaterials modeling, bedding for cells, and growth factor and drug delivery systems [18]. Collagen, elastin, laminin, and fibronectin are among the central AM's ECM constituents that would provide great scaffolds in RM processes, including wound repair [19]. To allow autologous/allogeneic grafting, DAM's stromal proteins may foster the anchoring and growth of several cell lines. In this realm, the transfer of autologous keratinocytes to an ulcer site after a full-thickness burn is one instance of how such a biocompound might be used as a carrier to treat various injuries [20].

The DAM has established itself as a highly adaptable biomaterial in RM, offering diverse applications due to its unique biological properties. Incorporation of cytokines and growth factors has significantly broadened its useful, particularly as a three-dimensional (3D) scaffold for TE and an innovative platform for drug delivery. Recent investigations have highlighted its remarkable drug delivery capabilities, demonstrating the sustained release of bioactive molecules such as cefazolin and moxifloxacin for up to 7 weeks [21]. Additionally, DAM has been mineralized with calcium and phosphate using the double diffusion method, creating membranes suitable for bone regeneration [22]. DAM provides a biocompatible ECM that supports cell culturing while preserving cellular properties, making it particularly effective in periodontal tissue regeneration. For example, DAM loaded with adipose-derived stem cells (ADSCs) has shown potent anti-inflammatory effects and accelerated wound healing in burn models [23]. Similarly, dehydrated DAM loaded with genetically modified TGF-β3 bone marrow stem cells (BMSCs) has significantly reduced scar formation and enhanced cosmetic outcomes in full-thickness wounds [24]. These advancements emphasize the DAM's potential as a rich biomaterial for tissue regeneration and a promising tool for sustained drug delivery systems. Despite its benefits, DAM-based scaffolds face challenges such as rapid biodegradation, suboptimal mechanical properties [10], and variability in drug release profiles [21]. The decellularization process can sometimes compromise ECM integrity, affecting scaffold performance. Furthermore, the scalability of DAM preparation and its storage requirements (e.g., cryopreservation) remain significant obstacles [25].

However, AM use dates back over a hundred years ago as a simple layer for intralesional administration for dermal and ocular surface healing [26]. This article discusses the evolution of the DAM and nanotechnology's contribution in paving the way for its increased deployment in TE and therapeutic settings. Recent advances in isolating AM's extracellular vehicles (EVs) and nanomaterials, NPs-loaded AM, and morphologic design modification, including cross-linking, have all accelerated its use in TE, jumpstarting the regenerative processes [27]. This publication summarizes recent progress in translating the DAM from laboratories toward clinics, providing an inclusive picture of the potentials of the isolated and enriched cell- and drug-containing scaffolds for translation into the clinic [28]. To further improve the DAM's efficacy, we want to study the latest innovations in postpurification DAM modification techniques, emphasizing cross-linking strategies.

This literature review does not include a dedicated methodology section for primary data collection, as its primary aim is to synthesize and analyze existing research. Nevertheless, to improve transparency and provide a clear framework for the selection of sources, a brief overview of the search strategy employed is presented. The literature search was conducted across several reputable databases, including PubMed, Scopus, and Web of Science. Keywords such as “amniotic membranes,” “decellularized,” “regenerative medicine,” “drug delivery system,” “tissue regeneration,” “scaffolds,” and “biocompatible materials” were used to identify relevant studies. Inclusion criteria were established to ensure the quality and relevance of the selected literature; these criteria comprised peer-reviewed articles published in recent years, with a particular focus on studies addressing combined scaffolding and drug delivery systems in TE.

## 2. The ECM Takes Center Stage by Decellularization

Unviability for storage, transit, and antigenicity makes fresh AM deployment challenging in therapeutic settings. Epithelial inclusion cystic lesions are commonly described as indicating AM graft rejection [29]. Decellularization is performed to eliminate all cellular components while protecting the biophysical and metabolic integrity [30]. To further explore, AM epithelium cells lessen the crosstalk between the cultivated cells and the stromal growth factors. Epithelial cell removal while sparing the AM's architecture improves biocompatibility [31]. Consistently, scholars seeded mesenchymal cells on intact AM and DAM, witnessing more robust differentiation to adipogenic lineage and formation of modular osseous tissues by osteogenic differentiation among DAM cultures, compared to AM. Hence, it is conceivable that DAM orchestrates the SC specification, providing superior support [32].

ECM is constituted of a constellation of proteins, glycoproteins, and growth-modulating bioagents [33]. In virtue of the tremendous ECM's intricacy, from the ground up, synthesis of ECM is not conceivable; hence, harnessing the features of decellularized tissues is growing in popularity as an alternative tool in RM [34]. DAM preserves several of the signaling biomolecules seen in human AM, including TGF-β, bFGF, IGF-1, VEGF, PDGF, and tissue inhibitor of metalloproteinase to choreograph damage healing, growth, and adherence [35]. Indeed, this membrane serves as an ideal substrate for cell cultivation. Decellularization could be accomplished by multiple techniques. The most beneficial approach should exert low levels of ECM changes while extracting the cellular components. Of note, decellularization methods are vastly described elsewhere [36] and are beyond the scope of this paper (Table 1).

Decellularization methods are pivotal in determining the suitability of DAM for applications in drug delivery and scaffolding. Among these, chemical and mechanical approaches using EDTA, NaOH, and NH_4_Cl have gained attention due to their effectiveness in weakening cell attachment to the ECM and inducing cell lysis. These methods are particularly valued for their cost-effectiveness, simplicity, and ability to preserve ECM components [43, 45].

The use of EDTA disrupts calcium-dependent cell adhesion, facilitating the detachment of cells from the ECM while maintaining ECM integrity [42]. NaOH, an alkaline agent, effectively lyses cells and removes cellular debris while preserving key basement membrane components like laminin and fibronectin. When combined with mechanical scraping or agitation, NaOH ensures thorough decellularization in a short time frame. NH_4_Cl contributes to cell lysis by disrupting osmotic balance within cells, further enhancing the decellularization process [46]. Together, these agents provide a biocompatible and efficient protocol for preparing DAM.

Enzymatic methods using dispase, thermolysin, and trypsin have been explored for decellularization. Dispase detaches cells but may damage ECM components if misused. Thermolysin preserves the ECM effectively, making it suitable for applications requiring minimal damage. Trypsin efficiently disrupts cell–ECM links but can degrade ECM proteins with prolonged exposure. Trypsin, a protease that breaks peptide bonds at the carboxyl end of amino acids, disrupts cell–ECM links efficiently. It demonstrates synergistic effects when used with other agents; however, prolonged exposure or high concentrations can degrade ECM proteins and growth factors [39].

Compared to chemical methods like SDS or Triton X-100, enzymatic approaches offer specificity in targeting cellular components but require careful optimization to avoid excessive ECM damage. For example, while SDS is highly effective in removing cellular remnants, it can compromise ECM integrity if overused. Similarly, Triton X-100 is gentler on ECM but less effective in complete decellularization [47, 48].

In terms of suitability for drug delivery systems, methods such as thermolysin or optimized trypsin protocols preserve ECM integrity while minimizing damage to growth factors, allowing DAM to act as an effective scaffold for sustained release of therapeutic agents. For scaffolding purposes, enzymatic methods like dispase or thermolysin ensure biocompatibility by retaining essential structural proteins required for cellular attachment and proliferation [49].

SCs loaded on DAM are brought into touch with the ECM, promoting SC growth and differentiation. Intriguingly, SC-DAM culturing helps study the genetic factors associated with SC maintenance and therapy [50]. Francisco et al. conducted in vivo research using DAM in place of pericardial tissue. Immunohistochemistry evidence suggests that DAM is a promising treatment option for pericardial pathologies since it fuses with the severed pericardium, reduces fibrosis and sores, increases pericardial density, and has a modest immunological profile [51]. Minjuan et al. found that nude mice healed third-degree dermal ulcers faster when adipose-derived mesenchymal SCs (AD-MSCs) were cultured on the DAM [52]. Congruously, Chen et al. demonstrated that apical papilla cells (APCs) might be driven toward an osteogenic lineage in the presence of DAM [53]. Cross-linking is considered an optimization process to improve the DAM to maintain the most advantageous principles of the native ECM. In the following sections, we debate the novel insights toward a cross-linkers role in DAM translation into RM [54].

## 3. Cross-Linkers Enhance the Efficacy of DAM in RM

The collagens are essential to creating mechanical resistance and resistance to hydrolysis. One of the most critical problems facing AM in soft tissue engineering is its low mechanical properties and rapid degradation [10]. The peptide bridges in the collagen nanofibers of DAM stabilize the ECM [55]. Following tissue damage, significant levels of highly activated collagenase are released, causing the biodegradation of AM by deteriorating these collagen bridges [56]. To increase the resistance of tissues and dampen the destruction rate, a range of chemical and physical cross-linking approaches have taken center stage in this context. Physical cross-linking methods improve the mechanical stability and strength of AM [54]. These methods involve exposing the AM to ionizing radiation, such as ultraviolet (UV) or electron beam radiation. Ionizing radiation can cause cross-linking of the molecular structure of the AM, improving its mechanical properties by increasing the intermolecular bonds between the proteins in the membrane [10]. The degree of cross-linking can be controlled by adjusting the dose and energy of the ionizing radiation. This method is considered a physical cross-linking, as it does not involve chemical agents. It is a straightforward and fast approach to improve the AM's mechanical traits for various medical applications, such as wound healing, skin replacement, and drug delivery. However, it is essential to note that ionizing radiation can also adversely affect the biocompatibility of AMs, such as inducing oxidative stress and damaging the membrane structure [57].

On the other page, chemical methods comprise various chemical reagents, such as glutaraldehyde (GTA) [58], genipin (GP) [59], 1-ethyl-3(3-dimethyl aminopropyl)-carbodiimide (EDC) [56], and aluminum sulfate [60].

Chemical cross-linking methods offer strong and stable bonds, making them effective for enhancing mechanical strength, but they can be cytotoxic and may alter the biological properties of the scaffold [61]. Physical cross-linking is generally nontoxic and can improve mechanical properties without the need for chemical additives; however, it may require specialized equipment and is often less effective in achieving long-term stability compared to chemical methods. Biological cross-linking is biocompatible and often bioactive, enhancing biological integration and healing, but it is typically less mechanically robust and can be expensive and complex to produce [61].

**Figure 1:**
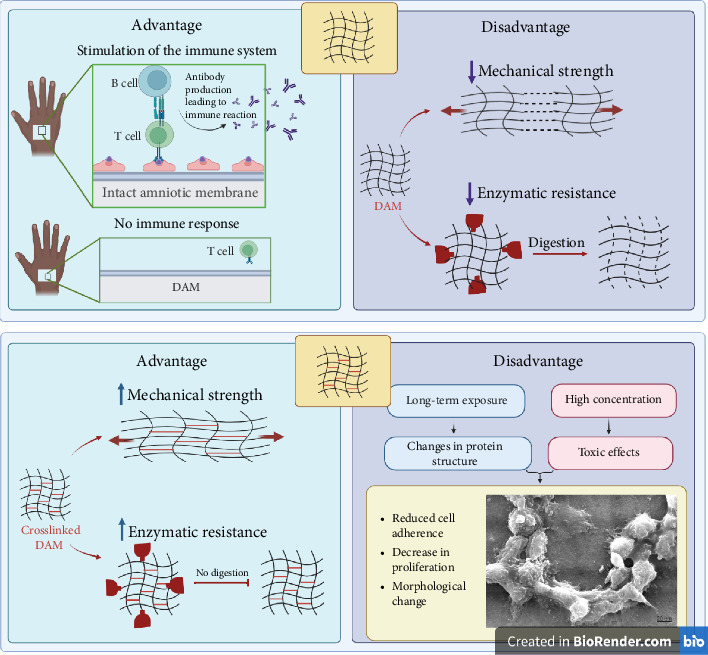
Decellularization prevents immune reactions. Cross-linking between collagen fibers increases mechanical and enzymatic resistance. The use of nonoptimal concentration and cross-linker exposure time reduces cell adhesion and proliferation. Created with https://www.BioRender.com.

In the case of AM, the cross-linking process can reduce the risk of membrane degradation and shrinkage, minimizing the risk of inflammation and scarring. Additionally, the chemical or physical modification of the membrane can improve its mechanical properties, making it a more effective transplant material [10] (Figure 1).

## 4. The Attributes of TE Scaffolds

Scaffolds assist cellular proliferation and stimulate and guide restoration to create fully fledged tissue during the repair process, virtually identical to how a fibrin clot supports tissue reconstruction in physiologic repair [62]. Scaffolds can be classified as synthetic, composite, or natural [63]. The former two categories could be mass-produced and controlled for resilience, membrane integrity, and disintegration. Natural scaffolds, however, appear more beneficial than synthetic or composite scaffolds, which are linked to issues including high cost, production of hazardous chemicals, limited biocompatibility, and immunogenicity [64]. Notwithstanding, although organic components seldom ever come from human origins, restricting their therapeutic complying, artificial composites typically lack the requisite architectural intricacy, such as a capillary bed to interact dynamically with the host's vasculature. Therefore, human-based RM compounds are being developed [65].

Current scaffolding materials in TE face limitations in mechanical properties, biocompatibility, degradation rates, and cell attachment. Natural polymers like collagen and gelatin are biocompatible but mechanically weak, while synthetic polymers such as PLA and PCL offer better strength but need surface modifications for improved cell attachment. Natural materials often degrade too quickly, compromising structural integrity, whereas synthetic materials can cause local acidosis and inflammation upon degradation [65]. Hybrid scaffolds, combining natural and synthetic materials, aim to leverage the strengths of both, enhancing biocompatibility and mechanical properties. Emerging technologies like 3D bioprinting offer precise control over scaffold architecture but face challenges with material suitability. Overall, hybrid and composite materials, as well as advanced techniques, hold promise for addressing these limitations [66].

Scaffolds composed of ECM-derived components have been used to enhance the reconstruction of various tissues [67]. ECM-derived scaffolds imitate the physiological milieu and may control multiple cell processes to stimulate restorative pathways by incorporating diverse cytokines and mechanical support through tissue repair [68]. TE relies heavily on scaffolds' customized construct and biocompatibility, with cell cultivation onto the nanostructures being the initial stage in forming a stereoscopic condition medium, thus contributing to the progression of tissue repair [69]. Cells, their origin, and the nanocomposite's fabrication design all have a role in whether or not the scaffold is successfully seeded with cells [70]. The scaffold's biocompatibility allows cells to adhere, migrate, and expand, similar to the physiologic environment [71]. Both the composition and geometry of a scaffold play a role in its ability to integrate with living tissue [72]. Biocompatible scaffold design is carried out through several stages. Scaffolds are designed with a porous mesh that allows nutrients to reach cells, remove toxic substances, and allow ECM production and vasculogenesis [73]. Furthermore, the scaffold must support consistent cell dispersion, development, and proliferation. The scaffold and its breakdown products should be nontoxic, a key property, as toxicity leads to inflammatory responses and graft rejection [74]. Immune tolerance is another critical attribute of a biocompatible scaffold. Notably, ECM replacement by scaffold cells is biodegradable [75]. Tissue formation should outpace scaffold degradation, which defines its life span. Consistently, the scaffold's mechanical qualities should mimic the physiologic milieu [76]. To serve as a tissue substrate, it should possess plasticity, elasticity, flexibility, and permeability [77].

From another perspective, traditional 2D cell cultures have drawbacks like a lack of ECM and cellular interconnectedness, constraining cell growth on a flat plane, which is not representative of the homeostatic condition, which features cell–ECM interplay as well as a gradient of micronutrients and cytokines [78]. 3D cell culture designs, including scaffolds and scaffold-free bioprinting, have developed in this context. Either through straight seeding of cells or by scattering cells in a fluid medium, polymerizing and solidifying, scaffold-based cultures are developed. Agitation-centered systems, hanging drop strategy, and forced-floating settings create scaffold-free spheroid suspension condition medium [79]. The nonscaffold cultures are beyond the scope of this paper.

## 5. AM Designated as a Versatile RM Tool

### 5.1. AM History and General Attributes

**Figure 2:**
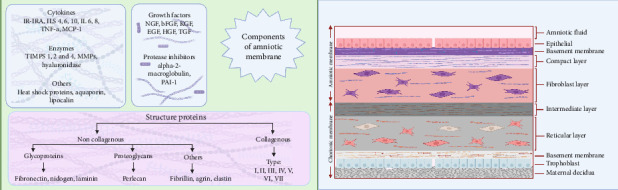
Amniotic membrane structure and components. Epithelial and mesenchymal cells of the amniotic membrane are responsible for the production of cytokines, growth factors, and protein structures. Created with https://www.BioRender.com.

AM is a transparent biomaterial in the innermost lining of embryonic membranes, accounting for the thickest body membranes (20–500 μm) [7]. The AM contains no neuronal, muscular, or lymphatic tissues. Three cell types make up AM: fibroblasts, mesenchymal SCs (MSCs), and amniotic epithelial cells (AECs). These cells possess antifibrotic, inflammation-resolutive, and significant immune-tolerant profiles, with angiogenesis-promoting, and oxidative burden-suppressing properties, due to hyaluronic acid, β-defensin, and elfin secretion and TGF-β reduction [80]. AM encompasses three compartments, which are the epithelium, basal lamina, and stroma, which the latter could be further broken down into an acellular compact lamina packed with reticular fibrils, a fibroblastic layer containing fibroblast cells, and a very hygroscopic spongy shit with fibrils interconnected to the chorion and the embryonic sac [81]. The epithelial layer, directly facing the fetus, is arranged in a unique homogenous sheet of cuboidal cells, characterized by epithelium-specific surface antigens, including pan-CK, CA 125, Muc 16, EpCAM, CDH1, and CD73. Besides producing and releasing ECM, epithelial cells might have contributed to the AM's poor antigenicity by expressing fewer MCH II markers. The specific structure of the cytoskeleton, such as actin, vimentin, cytokeratin, desmoplakin, and α-actinin in this layer, plays an essential role in maintaining its structure and permeability [82]. Heparan sulfate proteoglycans and collagens (I, III, IV, V, and VII) are abundant in the basal lamina, giving tensile strength and mechanical properties to AM. Laminin and fibronectin in this layer contribute to cell differentiation and adhesion, respectively. Accordingly, this membrane has lamina densa that is connected to the AECs basolateral surface integrin [83]. Ultimately, the stroma comprises compact collagens (I and III) and some strands of collagens (II, IV, and V) arranged in parallel to enhance tensile strength, which is secreted by the AMSCs dispersed in the fibroblast layer. It is followed by the fibroblast sheet, which contains reticulin, fibroblasts, and plenty of fibronectin and laminin and is dispersed by AMSCs, derived from the extra-embryonic mesoderm. Followed by the spongy layer, which encompasses proteoglycan and glycoproteins, thus appearing spongy in histologic examinations, and nonfibrillar meshwork of type III collagen, separating the AM from the chorion [84] (Figure 2).

Historically, Davis et al. first employed the amnion clinically in 1910 [85]. Kim et al. treated ocular disorders with amnion a century later [86]. After that, the amnion has found several medical applications, including exploiting its therapeutic impact on burn [87] and DFU [88], the rebuilding of damaged bladders [89], the healing of oropharyngeal sores [90], ocular recovery [91], and many more procedures. Additionally, owing to its superior qualities, which are discussed in greater detail in the following passage, AM would play a progressively prominent role as a biological scaffold and biocarrier in RM [92].

Recently, advancements in RM have expanded its applications. Decellularization techniques have enabled the creation of decellularized amniotic membrane (DAM), reducing immunogenicity and making it suitable for use as a scaffold in TE [75] for cartilage [93], bone [94], and nerve regeneration [95]. AM is also being explored as a drug delivery system [96], releasing therapeutic agents to enhance tissue repair. Additionally, its integration with SC therapy provides a supportive environment for SC proliferation and differentiation, improving treatment efficacy [48]. These advancements highlight AM's versatility in modern RM, beyond its traditional uses in wound care and ophthalmology.

### 5.2. The Acellular AM as an SC-Friendly Scaffold

**Figure 3:**
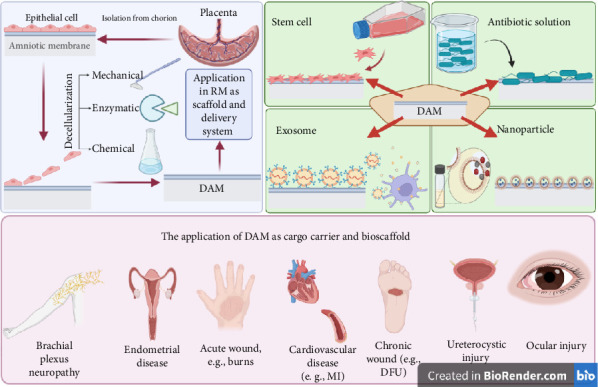
Decellularization of the amniotic membrane is done by mechanical, enzymatic, and chemical methods. The DAM is a viable scaffold for the targeted transfer of biological cargo such as antibiotics, nanoparticles, stem cells, and exosomes. The DAM carrying various therapeutic agents has shown great promise in treatment of an array of diseases. Created with https://www.BioRender.com.

SC therapy is well recognized as a powerful method for treating a wide range of medical conditions and traumas to tissues [97]. Local, intravenous, and intra-arterial administrations are all viable options for SC transplant [98]. Howsoever, the amount of SC in the target site may fail to reach the therapeutic level, along with the increased occurrence of hemorrhagic and thromboembolic events [99]. In this regard, the versatility of DAM as a bio-scaffold, which ranges from a monolayer composite for SC proliferation to further complicated 3D scaffolds, has garnered attention [36]. In recent decades, DAM has been extensively used as a biological transport matrix for cell-based treatment. Because of its substantial ECM content, accessibility, competitive prices, and extended storage capacity, DAM provides an exceptional intrinsic 3D scaffold for cell cultivation experiments (Figure 3). AM-derived scaffolds may supply ECM for cell development as a replacement for artificial composites [100]. However, some researchers argue differently, as Sharifiaghdas et al. cultured urothelial and smooth muscle cells on AM, collagen, and collagen/poly(lactic-co-glycolic acid) (PLGA) composites. They reported that despite the more pronounced interaction with the seeded cells, the AM scaffold matrix was weaker in terms of mechanical resistance than the other two scaffolds. In addition, according to the results of electron microscopy and MTT tests, the highest and lowest levels of cell adhesion and survival rates were found in the collagen/PLGA scaffold and the AM scaffold, respectively [101]. A plethora of research denotes the opposite, as AM-SCs are accountable for synthesizing mediators, growth factors, and structural polypeptides that contribute to ECM creation [6]. As outlined earlier, DAM comprises fibronectin, laminin, elastin, proteoglycans, hyaluronic acid, collagens I, III, IV, V, and VII, FGF-β, EGF, and TGF-β, along with numerous active compounds [102]. DAM scaffolding not only provides a supportive environment for cell development and division but also shields the site from contamination [103]. DAM-SC composites reach the targeted location with minimal interference with the surrounding tissue's homeostasis [104]. 3D-DAM scaffolds feature ECM constituents, polarity, and dynamic intercellular and cell–ECM signaling that 2D conditions lack, which may improve SC development more robustly [105]. Scholars have concreted the essential role of the ECM in SC development, evidenced by the integrin-mediated cell–ECM interplay, affecting the cell's transcription profile, cytoskeleton architecture, proliferation, motility, and maturation rate [106].

#### 5.2.1. In Vitro Studies

Dorazehi et al. examined the influence of CSF, as a great reservoir of neurotrophic agents, on MSCs isolated from bone marrow (BM-MSCs) and cultivated on DAM. They witnessed a promotive impact on neuronal specification of MSCs by the DAM and CSF concomitantly. This study demonstrated that BM-MSCs growth and viability are enhanced by DAM for up to 2 weeks. Although, in the fourth week, MSCs' survival diminished partly due to an alternate in the DAM composition, resulting from the buildup of cells' toxic biomaterials [107]. Intriguingly, Liang et al. discovered that the BM-SC-loaded DAM might promote cortical neurons' neurite growth. They showed that BM-SCs loaded on DAM preserved a remarkable survival level (> 85%). The mice cortex neurons cocultured on the BM-MC-DAM meshwork exhibited elongated NF-H positive axonal growth, compared to the isolated BM-MSC group (*p*=0.01), due to the released neuron growth factors and altered ECM. They concluded that DAM proved an excellent biocarrier for BM-SCs proliferation, and the BM-SC-DAM network showed promise for cortical neuron axonal outgrowth in vitro [108].

The use of EDC cross-linked DAM in limbal epithelial cell (LEC) culture was initially studied by Ma et al. Their research unraveled that the development of cross-links in the extracts exposed to 0.05 mmol EDC/mg AM may generate a considerable accumulation of tropocollagen biomolecules polymerizing into collagen fibrils with minimal impact on cell homeostasis. EDC at the mentioned dose may improve the scaffold's physical traits, translucency, and collagenase resistance. Also, cell development on DAM would benefit from albumin permeability via cross-linked AM. In vitro study demonstrates that EDC-cross-linked DAM samples supported LEC growth and epithelial progenitor cell maintenance. The optimal 0.05 mmol EDC/mg AM could pave the way for more concrete DAM applications in RM [56]. For cancer research, Ganjibakhsh et al. designed a 3D model employing a DAM composite. Cancer remains a leading cause of mortality, necessitating advanced research methodologies. The research utilized time-lapse imaging to analyze cancer cell behavior, focusing on proliferation, migration, and response to cisplatin treatment. Results indicated that cells in the DAM scaffold exhibited distinct behaviors compared to those in 2D cultures, including increased resistance to apoptosis and enhanced cancer SC (CSC) content. This suggests that the DAM scaffold is a promising tool for more physiologically relevant in vitro cancer studies, potentially improving therapeutic strategies [96].

#### 5.2.2. In Vivo Studies

Sang et al. evaluated the DAM's protective impact on tenocytes in vitro and in vivo. DAM retains the natural growth factors, such as TGF-β1 and bFGF, whose stimulatory effect on tendon cell differentiation and maturation has been established [35]. Its prevalence of collagen content bolstered the DAM's tensile properties, and the 3D porous meshwork provided enough room for tenocyte growth. Indeed, DAM is reminiscent of physiologic connective tissue, ergo, exerting a seminal affinity for tenocytes. Through the secretion of TGF-1 and bFGF, DAM triggered the rapid expansion of tenocytes with relatively static characteristics in vitro. To further cement their notion, they built a chicken tendon damage experiment to study the implications in vivo [35]. By measuring tendon attachment and staining fibroblasts, tenocytes, and monocytes with vimentin and CD68, the experiment showed how the DAM helps endogenous reconstruction while blocking exogenous repair. Vimentin is a biological marker widely employed because of its involvement in cellular viability, motility, and cellular interaction. Macrosialin (CD68) is a transmembrane protein highly expressed by cells in the monocyte lineage. The fibroblasts, tenocytes, and macrophages involved in tendon healing were stained with vimentin and macrosialin. In fact, IHC evaluations revealed a robust expression of these markers in the DAM-treated group compared to the controls. The vimentin's arrangement lent further credence to their observations as it displayed a multilayered and dense pattern, unlike the disordered composition of vimentin in the control group. Vimentin's flawed or aberrant placement may reduce tendon cell and fibroblast motility, hamper tendon repair, alter physical qualities, and compromise endothelial integrity. As a vital source of TGF-1 and bFGF, DAM helps endogenous repair by tenocytes and halts exogenous repair. This opens up new ways to treat restrictive tendon adhesion [35].

Kim et al. examined the potency of the MSC-DAM delivery system on rabbit models of skin defects compared to MSC's local injection. Researchers assessed the wound surface at different time intervals for each group, followed by a tissue repair quality assessment by IHC examination of the wound sections. An exuberant proinflammatory response was detected in the former group, with promising ulcer size reduction, while a dearth of locally injected MSCs survived on the target area post-injection. IHC results from the MSC-DAM group disclosed a fine epidermal layer with high scales of maturity, including organized structural fiber deposits and adnexa of skin embedded into the dermis. Contrariwise, the other group's IHC results manifested a gross epidermal layer with signs of immature epithelium, such as substantial fibroblastic invasion and minimal skin appendage development [109]. Aghayan et al. cultivated AD-MSCs and placenta-derived MSCs (PL-MSCs) on DAM for a week and transferred the composites to 24 full-thickness wound rat models. After 2 weeks of administration, the control group demonstrated strands of epithelial differentiation scattered in the wound area and some levels of inflammation. However, the epithelial cells yielded more robust proliferation in the therapeutic groups. Contrary to the dermis layer, which failed to show meaningful thickness changes. Signs of inflammation had abated, as evidenced by lower levels of PMNs and tissue macrophage invasion compared to the controls. In fact, compared to controls, they witnessed a hastened level of collagen synthesis and blood vessel remodeling and enhanced epithelium formation in AD-MSC-DAM and the PL-MSC-DAM groups. Compared to AD-MSC-DAM, the PL-MSC-DAM composite exerted more promising overhauling in the target site, evidenced by the reformation of skin appendages and pronounced interdigitations of the epidermis and dermis (rete pegs) [110]. Ebrahimi et al. seeded macrophages exposed to mesenchymal cell culture supernatants on DAM. Then, they transferred the activated macrophage-loaded DAM onto the rodent model of full-thickness wounds. The macrophage transcription profile studies yielded high levels of TGF-β, MRC1, ARG1, and IL-10 mRNA expression, implying the anti-inflammatory response they are inducing since these factors are highly involved in wound repair, tissue fibrosis, and local depletion of proinflammatory T cell activation. Also, researchers unraveled the plasticity and magnificent integration of the macrophages into the DAM meshwork. Transferring the fabricated scaffold to the wound site held promise in terms of abating the inflammation and accruing vascular and collagen deposition and remodeling [111]. Zhao et al. had previously demonstrated that physical outstretch (POS) entailed dynamic changes in the pelvic fascia's fibroblast (PFF) shape and expression profile. Also, provided coculturing with these POS-PFFs, BM-MSCs swayed to a pelvic ligament fibroblast morphologic and expression behavior. This contention is cemented by observing their transcription profile, which exerted a significant load of elastin, collagens (I and III), and genes associated with ECM formation and maturation, such as FBLN5 and lysyl oxidases. They continued their venture by applying the outlined cells onto the DAM bio-scaffold and evaluated its efficacy on mouse pelvic floor muscle dysfunction (PFMD) mouse models. Interestingly, by cell viability assay (CCK-8) and western blot, they established the beneficial contribution of DAM on the BM-MSCs expansion and integration into the DAM and ruled out its effect on BM-MSCs apoptosis. Investigations indicated that BM-MSCs grew radially, swirled, or in parallel a week after seeding on DAM. There were several extensions from the hypertrophic cytosol, mostly intertwined to form a network. The cell body itself displayed an asymmetrical, elongated, and pyramidal morphology. The DAM also held the cells firmly. RT-qPCR underlined the high scales of elastin, lysyl oxidase, and FBLN5 production in BM-MSCs cocultured with POS-PFFS loaded on DAM. The researchers concluded that the PFMD rodent models treated with the mentioned DAM composite rendered a better urodynamic profile, including higher bladder volume and a rise in VLPP [112]. The study conducted by Hariastawa et al. aimed to evaluate the effect of using an isolated, DAM scaffold and the DAM harvested with AD-MSCs in penile repair on rabbits. A month post-urethral defecting procedure, the penile epithelium healing was analyzed in different groups. The group with isolated DAM transfer to the lesioned penis demonstrated urethral amendment in 20% of cases, while the majority (80%) exerted penile urethrocutaneous fistulae (PUF) [113]. Rigorous healing was observed in the AD-MSC-DAM group yielding robust urothelial integrity in 75% of cases, although 2 out of 8 developed PUF, evidenced by a Fisher's exact test (*p*=0.029). The results unveiled the promoting impact of AD-MSC-DAM composite on the formation of normal penises without any fistulas. The reconstruction process maintained steady progress, without protracted swelling of adjacent tissue, inflammation, and cicatrix. In conclusion, the study suggests that stem the composite may provide a therapeutic avenue for urethral healing in male rabbits, but further research in human subjects is needed to validate these findings [113]. To explore the effect of intact amnion, DAM, and epithelial-to-mesenchymal transition (EMT)–induced AM by TGF-β construct on myocardial infarction (MI), Roy et al. performed LAD ligation in mice to provide a model of MI. Consequently, distinct AM types were stitched to the left anterior ventricles. In vitro, they observed a substantial EMT in AM following exposure to TGF–β, however, with the price of accrued immunogenicity both in culture (splenocyte expansion) and in mice (T helper preponderance) [33]. Additionally, IL-6 was released at a rate three- and one-hundredfold greater than IL-10 in vitro from intact amnion and cardiac fibroblasts cocultured with it, respectively; however, no biofactor release was detected from DAM. A month post-operation, they observed milder necrotic areas in mice with intact amnion and DAM administration, while the fibrosis was more prominent than the sham group [33]. They witnessed minimal variances in arteriolar preponderance and apoptotic bodies across the groups. The inotropic state of the heart was measured as most potent in intact amnion and EMT-AM mice, while the LV pressure and stroke volume were highest in mice taking DAM patches. According to the research, using DAM was proven to improve cardiac function. There is room for future investigation into the possibilities of this cell-free technique to assist the heart following an infarct [33]. Other scholars exploited AM with minimally remaining cells shaped into a cylinder with the basement membrane surface facing inward. These cylindrical structures were then inserted into the rodent-severed sciatic nerve (SSN) model. After 3 months, the researchers observed a substantial improvement in the repair of the SSN, with the cylinder being filled with neuronal tissue and showing signs of new blood vessel formation. Additionally, the number of neurons in the sciatic nerve of rats that received the DAM treatment was comparable to that of the rats treated with autografts, which is considered the benchmark for neural repair [114] (Table 2).

#### 5.2.3. Clinical Trials

Soetomo et al. conducted a nonrandomized clinical trial to evaluate the promise of an allogeneic AD-MSCs seeded on DAM bioscaffold to enhance the healing rate in severed supraspinatus tendon (SST). SST is the most commonly involved tendon in rotator cuff injuries. However, the widely utilized surgical methods, even the recent deployment of suture anchoring modalities, have failed to show promise in recurrence, particularly in large tears. Thus, it is justifiable to attempt to explore viable alternative therapeutic avenues. They investigated 22 individuals undergoing arthroscopic surgery for the SST injury and transferred the engineered composite to the target area. Their investigations denoted that a year following the surgery and bio-scaffold treatment, 91% of afflicted individuals showed SST repair, which MRI results corroborate. All cases affirmed prominent pain relief and function recovery, as established through VAS and CMS scores at 6 and 12 months post-surgery. Thus, developing TE strategies, harnessing SCs, and designating microenvironments with prominent loads of cytokines and growth factors could light the tendon repair outlook (NCT04670302). Inflammatory processes and iatrogenic manipulations might lead to endometrial adhesions and fibrosis, as in Asherman's syndrome, which substantially reduces fertility. The success of SCs in RM ushered in a new era of therapeutics based on cell delivery systems.

Harzif and his team designed an SC-DAM delivery system to help uterine endometrium recuperate to its physiologic state with proper menstruation. Firstly, they harvested patients' endometrial cells (ECs) with amniotic epithelial SCs (AESCs), as evidenced by CD54, BRCP1, and T cell receptor alpha locus expression, CD15, Oct4, and Nanog, respectively. Next, they would inoculate the EC-AESC cocultured into the DAM meshwork. The researchers hypothesize that the outlined construct could facilitate endometrial recovery (NCT04676269). Another prestigious study is a single-arm, open-label, phase 1/2a trial that evaluates the safety and feasibility of DAM concomitant with bone-patellar tendon-bone (BPTB) autografts for ACL healing. Although ACL repair surgery is one of the most common orthopedic surgeries, the state-of-the-art surgical methods for ACL tears take up to 1.5 years until complete recuperation, with a substantial risk of graft integration failure. To further explore, a synovial membrane to supply blood and nutrition physiologically coats ACL. Following damage, the latter is disintegrated, and the bottleneck of conventional ACL surgeries is the lack of this layer on the reconstructed surface, which hinders the integration and maturity of the graft. In this context, researchers exploited the AD-MSC-DAM composite in place of the bona fide synovial lining, concomitantly delivering SCs and growth factors. Among 19 participants, no serious adverse events related to the use of the DAM were reported. In addition, the preliminary results revealed that the AD-MSC-DAM application in ACL reconstruction was associated with improved graft maturation, as evidenced by MRI results. Specifically, the mean T2 relaxation time of the graft at 6 months post-surgery was significantly lower than the preoperative values, suggesting improved tissue maturation (NCT03294759). The clinical study designated as NCT04654286 is a phase 1/2a, open-label, single-arm trial focused on determining the safety and practicality of AD-MSC-DAM for enhancing functional recovery in patients with toe brachial pressure index (TBPI) who are receiving nerve transfer surgery. Although nerve transfer remains the standard treatment, its outcomes are frequently constrained by a lack of available donor nerve tissue and the peripheral nervous system's limited ability to regenerate after nerve injury. DAM represents an innovative RM strategy that utilizes advanced bioengineering to encourage nerve fiber regrowth and support the body's own neuronal repair mechanisms. If this trial demonstrates that the approach is both safe and beneficial, it could lead to a new treatment avenue for TBPI patients, with the potential to improve their movement (such as shoulder function), sensory abilities, and overall well-being. NCT04728906 is a clinical trial investigating using a heart patch composed of DAM cultivated with AESCs and patients' cardiomyocytes as a TE therapeutic alternative for MI. The heart patch is designed to regenerate damaged cardiomyocytes caused by the thromboembolic sequela of COVID-19, increasing its death toll. The study is a single-arm, open-label, phase 1/2 clinical trial that aims to evaluate the safety and efficacy of using the heart patch in patients who undergo bypass surgery (CABG). As of the latest update in January 2023, the study is ongoing and actively recruiting participants. If the study's results show that the heart patch is safe and effective, it could potentially contribute to novel therapeutic approaches for MI-afflicted individuals, particularly those with COVID-19 complications, which could improve their overall cardiac function and prognosis (Table 3).

### 5.3. DAM Surfaces Improve Distinctive SC Qualities

It has been shown that DAM affects the differentiation capacity and development of MSCs. Based on the DAM's fetal and maternal interfacing sides, MSCs cultured on it exert more adipogenic and osteogenic behaviors, respectively, than MSCs cultured on tissue culture plastic (TCP). The epithelial side of DAM differentiates soft tissues like fat and liver cells better than the stromal side, which encourages osteogenesis. Presumably, the varied behavior of MSCs on separate faces of DAM is owing to each side's distinct origin (fetal or maternal), development at distinct phases of fetus development, and ECM subassemblies [123]. On DAM application in osteogenic TE, MSCs derived from mice bone marrow (BM-MSC), human periodontal ligament (HPL-MSC), and human apical papilla (HAP-MSC) were tested for osteogenic differentiation. ALP level (a hallmark for pro-osteogenic signaling), osteogenic transcription profile (e.g., osteocalcin), and mineralization were all considerably greater in MSCs cultivated on DAM after 3 weeks in comparison to MSCs cultured on TCP [124]. A single layer of HPL-MSCs was deposited on DAM and subsequently implanted into mice's maxillary bone with periodontium defects. A month later, the mentioned mixture decreased the lesion compared to controls with exclusive DAM administration and boosted bone growth. IHC analysis revealed that DAM cultivated with HPL-MSCs produced significantly higher loads of cementum as opposed to the control group [125]. Chen et al. unraveled that the ALP levels in HAP-MSCs grown on either surface of DAM were greater than that of TCP regardless of whether or not the cells were exposed to an osteoinductive medium. Mainly, DAM's maternal surface adjacent to chorionic tissues is more potent at stimulating bone formation because ALP transcription, phosphorylated core-binding factor alpha 1 (CBFa-1), and mineralized deposition were recorded higher there than on the fetal surface [53]. Thus, insinuating that stimuli drive bone formation outside of cells; for instance, administration of a highly selective inhibitor of both MEK1 and MEK2 pathways dampened the AM-mediated osteogenesis. Future research is warranted to determine the associated factors, paving the way for DAM-mediated HAP-MSCs bone-forming application in RM [53].

Parenchymal designing in TE may benefit from the DAM's basal lamina surface because it stimulates the development of hepatocyte-like cells (HLCs) from MSCs isolated from adipose tissue. While HLCs developed on collagen I–covered controls were flat and had sharper margins, HLCs cultivated on the DAM's fetal surface were round, smaller, and featured numerous villi along with a greater transcription of MRP2, a liver cell hallmark [36]. HLCs cultured on DAM exerted higher transcription levels for liver-associated cytochromes, such as CYP3A4, CYP7A1, and CYP2B6, in controversy to those cultivated on TCP and collagen I. HLC-DAM matrix transplanted into rats with acute hepatic failure. Integrating into rat hepatic parenchymal tissue began by the end of the first week, and in 2 months, capillary formations were witnessed. Also, the phenotypically developed embedded HLCs could metabolize the test substance [36].

In the study of Sharifiaghdas et al., AM, collagen, and peritoneum scaffolds were used to culture mouse urothelial cells. They discovered that the basement membrane side of the DAM is more suitable for the culture of urothelial cells compared to the other two scaffolds [101]. Intriguingly, research has confronted contradictory observations on comparing the viability of the basement membrane side in DAM and the epithelial side in the intact AM about cultivating SCs. Some studies reported the basement membrane side in DAM [126], and others denote that the epithelial side in intact AM is more suitable for LSC [127] and keratinocyte culture [119]. So far, several studies have used the basement membrane side to culture fibroblast cells; their rationale is that it is a nutrient layer providing robust adhesion, growth, and proliferation of cells. Generally, epithelium in the intact AM is regarded as a physical barrier preventing the seeded cells from reaching the enriched milieu in the basement membrane and ECM [128]. In contrast, it was shown that the increase in the expression level of nerve growth factor (NGF) and limbal explants (K252a) at the epithelial level in intact AM, along with the growth factors present at the stromal level, causes better adhesion and growth of limbal cells. In vivo explorations unveiled that the stromal side of the AM reduces inflammatory responses and spurs angiogenesis by inhibiting the expression of IL-1. The benefits of using this side of AM are inhibiting TGF-β signaling and enhancing the proliferation and differentiation of the corneal myofibroblasts while hindering conjunctival surface fibrosis during the reconstruction [128]. In fact, the stromal side of the AM is regarded as a support system for the culture of rabbit chondrocyte cells [129], urothelial cells [130], and 3T3 cells (primary mouse embryonic fibroblast) [131].

## 6. Shades of Novelty in DAM Exploitation

During the last several decades, amnion applications have progressed from simple sheets for skin or eye healing to high-tech uses, such as amnion nanocomposite, powder, or hydrogel for tissue regeneration [6]. Nevertheless, adjustments are certainly needed given AM's intrinsic flaws, which include poor mechanical characteristics, short-term therapeutic effectiveness, manualing and stitching challenges, and significant lack of adherence [132]. To overcome the limitations of DAM, researchers suggested developing biocomposites based on DAM by incorporating polymers, fibrin glue, and other materials [6]. Instead, DAM may be employed in composite form to enhance another material's biological properties [133]. The creation of hydrogels derived from AMs is a recent area of research [134]. The state-of-the-art SC-centered TR techniques have limitations, such as minimal cell enrichment and transfer to target tissue, as well as inefficient post-grafting adherence. Owing to their safe profile and resemblance to the natural ECM, hydrogels are a desirable delivery system for keratinocyte and fibroblast cultivation and distribution [135].

The most popular method for improving AM qualities involves combining multilayered AM constructions with a polymeric layer made in various methods. One strategy is to conjugate a surface-activated nanofiber mesh [136] on top of an electrospun layer or directly electrospun the secondary material on top of the DAM [137]. Silk has found extensive use in RM thanks to its safe profile, minimal antigenicity, convenient handling, resistance to degradation, and outstanding biophysical characteristics. Silk nanofibers were occasionally electrospun DAM, particularly for skin regeneration. These constructions enhanced keratinocyte differentiation, cell adhesion, skin regeneration, and superior 3D structure maintenance. It was shown that DAM/silk composite had the same in vitro impact on cell survival and cytotoxicity as plain DAM [138]. Self-assembly and deposition are two methods by which biochemical substances are overspread on the DAM. Occasionally establishment of a biofilm on the ocular membrane upon DAM grafting could lead to severe infection. To fend off the infection, Mandel and colleagues covered DAM with clavanin A, an antimicrobial peptide, through self-assembly method to hinder fungal biofilm accumulation on the eye surface. The clavanin A-DAM complex was evaluated concerning biosafety, colonization and cell adherence in non-neoplastic cell lines (3T3 and human embryonic kidney cells). According to the outstanding structural, anatomical, and antimicrobial properties of the complex, clavanin A-coated DAM yield promise for managing ocular infection [137]. Another approach is to enhance the AM's antibacterial properties by electrospinning a layer and adding silver NPs.

Several studies appraised the properties and performance of DAM scaffolds as drug delivery systems (Table 4). Besides the duration of the loading process, it has been reported that AM thickness directly affects the loaded drug concentration and release kinetics in such a way that the thicker the AM is, the more drug entrapment it exerts [140]. Recent research demonstrated the extended-release kinetics of voriconazole from HAM, achieving sustained drug release for up to 5 weeks. This highlights HAM's potential as a reservoir for antifungal drugs in treating fungal keratitis, further supporting its suitability as a prolonged drug delivery system [150]. Many studies explored the loading of antiviral and antibacterial drugs on scaffolds. The findings suggest that, without sacrificing stability, DAM can be an appropriate drug carrier for the prolonged administration of enriched formulations [21]. Another study investigated whether the DAM's incubation with antiviral drugs can inhibit virus growth in laboratory conditions. The viral growth analysis revealed that compared to untreated DAM, the DAM group soaked in acyclovir or trifluridine effectively suppressed the expansion of herpes simplex virus (HSV) in cell cultures in dose-dependent manner [139]. Improved antibacterial properties were also seen in the AM impregnated with green silver NPs. Researchers created a nano-sized bioscaffold with a protracted shelf life for tissue regeneration by electrospinning umbilical cord collagen and green silver NPs on cross-linked DAM. Preparing the green silver NPs with curcumin using decreasing silver nitrate facilitated scarless healing because silver reduces the inflammation and curcumin promotes tissue repair. They showed that this dressing speeds up wound healing, offering a continuous, controlled release of silver while requiring fewer dressing changes compared to intact AM because of its substantially increased tensile strength [151]. Singh et al. inoculated the DAM with silver to create an antibacterial dressing. They showed the potential of DAM impregnated with silver to avert microbial infection and its notable physical characteristics for burn wound dressing [152]. Interestingly, the glitazone reverse insulin resistance receptor (NR1C3) is an inducible transcription factor with profound inflammation-modulating effects. 15-Deoxy-Δ12,14-prostaglandin J2 (15d-PGJ2) yields significant affinity for NR1C3, rendering it beneficial to dampen inflammatory response genes, such as iNOS and TNF signaling. Fransisco and colleagues witnessed the significant promise of DAM loaded with 15d-PGJ2 NPs in alleviating the ROS burden and inflammation in an in vitro model of chronic inflammatory disorder [153]. In an experiment on patients undergoing trabeculectomy, researchers exploited the DAM loaded with 5-fluorouracil PLGA NPs and demonstrated that the PLGA-DAM NP delivery system functions as an efficient antifibrotic therapeutic modality and improves surgery results in the long run [154].

### 6.1. Exosomes and miRNAs

Exosomes are also a potential delivery vehicle for wound healing promotion. Research has shown that exosomes derived from MSCs and ADSCs can promote wound healing, particularly in chronic diabetic lesions. ADSC-derived exosomes have been found to increase cell migration, proliferation, and collagen/elastin production while reducing scarring. However, the challenge is to find an effective and noninvasive way to use exosomes for therapeutic purposes. Using exosome-incorporated DAM scaffolds shows promise for enhancing diabetic skin wound healing and could be a potential treatment option. The DAM-Exos dressing accelerated wound healing in an in vivo diabetic skin wound model by controlling inflammation, improving vascularization, and encouraging ECM synthesis. Therefore, DAM, as an ideal scaffold for exosome delivery, offers compelling evidence for potential therapeutic uses of exosomes [124]. The study found that the dressing promoted the sustained release of exosomes, improving wound healing and tissue regeneration in diabetic mice. The results suggest that this approach can potentially develop an effective and practical therapeutic strategy for diabetic wound healing [124].

MicroRNAs (miRNAs) have been identified as crucial regulators of wound healing pathways, with the potential for targeted delivery of miRNA molecules in treating burn and diabetic wounds [155]. Upregulated and downregulated miRNAs have been identified in both wound types, with functions in regulating arteriogenesis, fibroblast, and keratinocyte expansion, microbial clearance, ECM remodeling, and scar modification [156]. Transfer of miRNA and anti-miRNA molecules presents a challenge, and investigation into delivery strategies is necessary [157]. Recent advancements in RNA delivery techniques have led to functionalized wound dressings or bioscaffolds transferring miRNA or anti-miRNA modulators in RM [158]. Smart light-activatable miRNA-loaded nanocarriers and polymeric NPs are two advanced systems for sustained delivery of miRNA molecules, which yield promise when accompanied by the conventional methods of designing skin grafts [159]. Intriguingly, we have noticed a gap in AM-based TE, as there is no published research on using miRNA-loaded AM or DAM in RM. Further research is warranted to explore additional AM-based delivery strategies for exosomes and miRNA-based therapeutics.

## 7. Conclusion

It would be reasonable to consider DAM as a potential allogeneic resource for RM, it serves as a substrate for autologous/allogeneic cell transfer and maturation and a scaffold for tissue repair, thanks to its various extracellular proteins enabling SC growth and the delivery of drugs and biologic agents choreographing tissue repair. The surface modification and cross-linking, design of AM-based nanocomposites and encapsulation with NPs, the development of amnion hydrogel, and micronization process have further enhanced the properties of AM matrix for regenerative applications. While manufacturing methods for DAM have advanced considerably, further studies are warranted to investigate their promise for TE thoroughly. Attempting to solubilize AM may produce more uniform surfaces for cell proliferation and biomaterials with tailored biophysical features, including injectable hydrogels. Furthermore, these membranes represent a vital resource for exploring cell–ECM interplay in physiological and pathological conditions, providing insights into the mechanisms governing cell properties such as maturation, proliferation, differentiation, and adhesion. In conclusion, the natural ECM of AM containing active molecules presents numerous promising applications in RM. A review of the current literature on this topic may serve as a valuable resource for clinicians and manufacturing companies.

Our comprehensive review of advancements in DAM highlights significant strides made in its application within RM. By collating and analyzing the latest research, our work provides a critical resource for researchers and clinicians aiming to understand the potential and limitations of DAM. This detailed examination of various decellularization techniques, biocompatibility enhancements, and integration with SCs can guide researchers in refining their methods and exploring new avenues in TE. Identifying current limitations and successful applications, our review highlights areas that require further investigation, helping to direct future research efforts. The exploration of DAM as a scaffold for drug delivery, customizable scaffolds, and 3D bioprinting opens up new possibilities for innovative applications that future research can build upon. Clinicians can leverage the insights from our review to implement more effective treatment protocols using DAM for chronic wound healing, orthopedic regeneration, and cardiovascular repair. By showcasing successful clinical trials and emerging uses, our work encourages the adoption of DAM in new medical fields, such as nerve regeneration and ophthalmic surgeries. The comprehensive analysis provided in our review can inform clinical decisions, helping practitioners choose the most appropriate DAM applications and techniques for their patients. The decellularized amniotic membrane (DAM), with its unique biocompatibility and structural properties, has become a pivotal material in drug delivery applications. However, it is worth noting that further studies are needed in this area, as current research is still incomplete and ongoing.

DAM exhibits remarkable biocompatibility, structural integrity, and the ability to sustain the release of therapeutic agents such as antibiotics, antivirals, and growth factors, as demonstrated in studies using cefazolin, moxifloxacin, and voriconazole. These properties make it a valuable tool in ophthalmology, wound healing, and cancer research. However, challenges persist, including variability in decellularization methods, donor heterogeneity, limited shelf life, and inconsistent drug release profiles. Addressing these issues through standardized protocols and advanced engineering techniques such as integrating NPs or developing micronized forms could enhance DAM's potential. Furthermore, its intrinsic properties, such as immunomodulation and promotion of epithelialization, provide a unique advantage for RM applications. Expanding on these aspects highlights DAM's transformative role in drug delivery systems.

## Figures and Tables

**Table 1:** Comparison (mechanism, advantage, and disadvantage) of various chemical and enzymatic decellularization methods.

Method		Mechanism	Advantage	Disadvantage	References
Enzymatic	Dispase	Disrupt cell ECM interactions and detach cells	—	As a protease, can damage collagen types IV and VII, fibronectin and laminin	[37]
	Thermolysin	Disrupt cell ECM interactions and detach cells	Effectively denudes AM with minimal destruction of basement membrane and ECM	—	[38]
	Trypsin	Breaking the peptide bond from the carboxyl end, disrupting cell–ECM links	Synergism with other agents	Degrades ECM proteins and growth factors in higher concentration or extended exposure time	[39]

Chemical	TritonX-100	It destroys lipid–lipid and lipid–protein interactions but does not damage protein-protein interactions	It does not change the structure of ECM and scaffold	Removes the GAG (glycosaminoglycan)	[40]
	Sodium dodecyl sulfate (SDS)	It causes cell lysis by destroying the cell membrane	Remove cells completely	Degradation of protein structure	[41]
	Urea	Detaches epithelial cells through solubilizing proteins	—	It may change the protein structure	[31]
	EDTA	Weakens cell attachment to ECM by chelating calcium and magnesium	Boosts activity of other agents, e.g., trypsin	Inefficient cell removal when utilized alone	[42]
	EDTA/NaOH/NH_4_Cl (chemical/mechanical)	Weaken cell attachment to ECM and cell lysis	Cost-effective and simple protocol Biocompatible Preservation of ECM compounds	—	[43]
	Per-acetic acid 2 M	Dissolves cytoplasmic compounds and destructs nucleic acids	Highest cell elimination and structure preservation	It removes the GAG	[44]

**Table 2:** A comprehensive assessment of in vivo and in vitro studies, which explored the therapeutic role of DAM in various pathologies.

Experimental settings	Culture system	Cell type	Therapeutic goal	Biological function	Conclusion (study results)	References
In vitro	Heterograft	Rat bone marrow mesenchymal stem cells (BM-MSC)	Neural tissue repair	Microtubule-associated protein 2 and beta-III tubulin were expressed in BM-MSCs cultured on DAM and treated with e-CSF	DAM effectively improves the BM-MSCs cultivation and differentiation	[107]
In vitro and in vivo	Heterograft	Rat BM-MSC	Bone defect repair	DAM shields the invasion of the fibrous tissues, stabilizing the bone grafts and inducing massive bone growth	Minimal toxic effects when cocultured with MSCs, as evidenced by high cell density, and osteogenic differentiation after 21-day culturing	[115]
In vitro	Autologous	HCE-2 cells, a human corneal epithelial cell line	Ocular injury repair	Carbodiimide cross-linking of DAM scaffold supports cell growth and survival	Carbodiimide cross-linked DAM is as a biocompatible scaffold for human corneal epithelial cell	[116]
In vitro	Heterograft	Rat BM-MSC and rat neural cortical cells (coculture)	Axonal outgrowth stimulation	BM-MSCs guide axonal outgrowth, through secreting neurotrophic factors and changing the ECM	BM-MSCs seeded DAM stimulate axonal elongation	[108]
In vitro	Heterograft	Rabbit chondrocytes	Severed cartilage repair	DAM increases the proliferation of chondrocytes, GAG expression, and GAG/cell interplay	DAM is a potential substrate/carrier for cell-based cartilage therapy and transplantation	[117]
In vitro	Autologous	Human smooth muscle cells	Vascular grafting	Smooth muscle cells adhere, proliferate, and exert remodeling on the scaffold during a 4-week culture period	The DAM vascular graft can be created with specified diameters and wall thicknesses to satisfy specific anatomic requirements in patients	[118]
In vitro	Autologous	Human keratinocytes	Traumatic wound repair	DAM modulates TGF-ß responses and accelerates chronic wounds' epithelialization	DAM enhances epithelialization on chronic wounds	[119]
In vivo	Heterograft	AD-MSCs and placenta-derived MSCs (PL-MSCs)	Wound healing	The wound closure rate, re-epithelialization, angiogenesis, and collagen remodeling were assessed	PL-MSCs seeded on DAM had superior regenerative effects to AD-MSc in the rat model of excisional wound	[110]
In vivo	Autologous	Rabbit BM-MSC	Wound healing	A thin epidermis with mature differentiation and deposition of collagen bundles along with recovered skin appendages was seen	The graft of DAM loaded with MSCs facilitates healing of skin defects in rabbits	[109]
In vivo	Heterograft	Rat BM-MSC	Pelvic floor dysfunction (PFD) repair	DAM can promote BM-MSC proliferation and differentiation into ligament fibroblasts	BM-MSC-DAM exert potential as a cell-based therapy for PFD	[112]
In vivo	Autologous	AD-MSC and fetal fibroblasts (FF)	Burn wound healing	Lower inflammatory cell infiltration was seen in AD-MSC loaded DAM and DAM-FF groups	Cell-based engineered skin substitutes accelerate wound regeneration	[120]
In vivo	Heterograft	Rabbit BM-MSC	Wound healing	Dermis depth, active collagen type I masculine cells and blood vessels increased	BM-MSC-loaded DAM encapsulated with bFGF and vitamin C renascences epidermis regeneration	[121]
In vivo	Heterograft	Macrophages	Incisional wound healing	Macrophages stimulated by MSCs supernatant improved healing by decreasing the inflammatory phase and fibrosis and increasing angiogenesis	Activated macrophages transplantation using DAM accelerates wound healing	[111]
In vivo	Autologous	Human BM-MSC and limbal stem cell (coculture)	Corneal defect	Induced BM-MSCs on DAM upregulated corneal stem cell markers; ß 1-integrin, C/EBPd, ABCG2, and p63, increased expression of CK3 and p63	BM-MSCs can be induced into corneal epithelial lineage used for corneal surface repair in a limbal stem cell–deficient model	[122]

**Table 3:** Comprehensive evaluation of clinical application of DAM bioscaffold in clinical trials.

Trial ID	NCT04676269	NCT04728906	NCT04654286	NCT04670302	NCT00736307	NCT03294759
Study title	Amnion bilayer and stem cell combination therapy on thin endometrium infertile patients	Heart patch for myocardial infarction COVID-19	Human amniotic membrane and mesenchymal stem cells composite	Mesenchymal stem cells and amniotic membrane composite for supraspinatus tendon repair augmentation	Autologous transplantation of cultivated limbal stem cells on amniotic membrane in limbal stem cell deficiency (LSD) patients	Bio ACL reconstruction amnion collagen matrix wrap and stem cells
Date	2019–2021	April 2022 to March 2022	2016–2022	2019–2022	2007–2009	2020–2023
Study design	Early phase 1, randomized, parallel assignment, quadruple	Single group assignment, open label	Nonrandomized, parallel assignment, single (outcomes assessor)	Phase 2, parallel assignment, single (outcomes assessor)	Phase 1 and phase 2 N/A, single group assignment, open label	Randomized, parallel assignment, double
Conditions	Infertility due to endometrial defect	Myocardial infarction	Brachial plexus neuropathies	Supraspinatus tear	Limbal stem cell (LSC) deficiency	ACL tear
Status	Recruiting	Recruiting	Unknown	Completed	Completed	
Maine effective path	Regenerate and recover the capability of the endometrial lining back into its cycles	Regenerating damaged cardiomyocytes	Augmentation of axonal regeneration	Biological augmentation in tendon repair	Corneal surface reconstruction	Reestablishing the natural synovial lining of the reconstructed ACL
Inclusion criteria	Ages up to 40 years, thin endometrium without a scar, acute thin endometrium post-therapy (medicaments)	Aged 40–60 years old, ischemic burden > 10% and ischemic gradients red-violet, ischemic area is not feasible to be grafted (bypass), ejection fraction (EF) > 30–35%	Ages 15–55 years, suffering from upper BPI for less than a year, without diabetes mellitus (DM), lupus erythematosus, rheumatoid arthritis (RA), without prior medicamentous treatment history such as corticosteroids	Ages 35–75 years, complete tear of the supraspinatus tendon for less than a year	Ages 18–75 years, patient with unilateral LSC insufficiency and total corneal vascularization, presence of goblet cells on the cornea, tear size > 5 mm, duration of deficiency > 3 years	18–45 years, patients undergoing ACL reconstruction with autologous grafts
Exclusion criteria	Patients with thin endometrium due to TB or cancer in the reproductive system	An ischemic area on Technetium-99 scan, undergoing other procedures, chronic kidney failure, immunocompromised patients	Complete BPI (C5-Th1), lower BPI (C8-Th1), traumatic BPI associated with delayed/nonunion fracture of the upper extremity affected side, poly trauma conditions which are not fully recovered	Patients with DM, RA, and other inflammatory diseases or other related injuries (fractures or dislocation around the shoulder joint)	A systemic disease affecting both eyes, such as Stevens–Johnson syndrome	Patients must be willing to undergo MRI scans postoperatively
Arms	Sham comparator: Amnion only, Experimental 1: Amnion–self-endometrium stem cells (EnSC), Experimental 2: Amnion–amnion epithelial stem cells (AESC), Experimental 3: Amnion–coculture self-EnSC—AESC (coculture)	*N* = 10 Experimental: Patients who undergo bypass (CABG) surgery are given heart patches in areas where grafting (bypass) is not feasible	*N* = 24 Control: The patient will receive a nerve transfer procedure without augmentation Experimental: Following the nerve transfer procedure, the end-to-end anastomosis will be wrapped with HAM-AD-MSC composite as augmentation	*N* = 24 Control: undergo tendon repair procedure only Experimental: undergo tendon repair procedure augmented with AAdMSC-HAM composite	*N* = 10 Experimental: Cultured limbal stem cells transplantation	Control: Reconstruction with either patella or hamstring autograft will be performed. Experimental: SCs isolated from bone marrow aspirate from the distal femur will be injected inside the amnion scaffold
Outcome measures	7 and 14 days after DAM seeded with endometrial cells cocultured with hAESC: Change in endometrium thickness and change in amenorrhea severity	Primary: Change of the ischemic burden (%) and regional heart wall motion abnormality Secondary: Change of electrocardiographic wave and EF	Primary: Active range of motion (AROM) pre-surgery and at 12 months follow-up, functional motor power outcome at 12 months follow-up Secondary: Initial elbow flexion MRC grade 1 (in months), initial elbow flexion MRC grade 3, disabilities of the arm, shoulder, and hand (DASH) score pre-surgery	Primary: AROM pre-surgery and at 12 months follow-up Secondary: Tear recurrence, DASH score pre-surgery	Primary: Snellen visual acuity, corneal epithelial integrity, and stability Secondary: Impression cytology, the extent of retarding recurrent neovascularization	Primary: Changes in ACL post-op, MRI region of interest mapping to produce mean T2 values Secondary: Changes in patient-reported pain rating post-op, changes in knee injury and osteoarthritis outcome score (KOOS)

**Table 4:** Information regarding the DAM's applications as a drug delivery system.

Cargo type	Specific agent	Study type or experimental settings	Target	Outcome	References
Drug	Cefazolin	In vitro	Corneal cells	AM may be a suitable drug carrier for extended delivery of fortified formulations without compromising stability	[21]
Drug	Acyclovir or trifluridine (antiviral)	In vitro	Monkey kidney cell line	Antiviral-treated AM as a drug-delivering tool inhibited viral replication in vitro	[139]
Drug	17b-estradiol (E2)	In vitro	Endometrial injury	The cell proliferation experiments showed that slow and even release of 17b-estradiol (E2) by the scaffolds is more conducive to the growth of endometrial cells than free E2	[40]
Drug	Moxifloxacin	Clinical	Infectious keratitis	DAM provided sustained delivery of drugs through a biological bandage	[140]
Drug	Ofloxacin	In vivo (rabbit)	Corneal epithelial defects	AM transplantation enhances ofloxacin penetration in corneas with epithelial defects	[141]
Drug	Ofloxacin	In vitro	Infectious keratitis	AM acted as an ofloxacin slow release device for up to 7 h in vitro, depending on the duration of pretreatment of AM	[142]
Drug	Netilmicin	In vitro	Infectious	AM can absorb the netilmicin and shows promise in antibiotic delivery	[143]
Nanoparticle	Silver nanoparticles	In vitro and in vivo	Wounds and burns	Long shelf life, easy application, sustained/controlled release of silver, provided minimal dressing changes, affects excessive exudates, moisture retainment, reduces inflammation and facilitates autolytic debridement. A promising dressing material for none healing ulcers and burns	[144]
Drug and nanoparticle	Colistin and silver nanoparticles	In vivo	Burn wound infected	Synergistic colistin and silver nanoparticle–loaded DAM enhances antimicrobial efficacy for burn wound treatment	[145]
Nanoparticles	Silica nanoparticles	Experimental	Vascular tissue regeneration	SiNP prevents the unraveling of a DAM while improving the scaffolds' overall mechanical properties	[146]
Nanoparticles	15d-PGJ2	In vivo	Post-infarct ventricular dysfunction	Facilitated colonization of fibrotic myocardium regions with new contractile cells and preventing reduction of left ventricle wall thickness.	[147]
Nanoparticles	15-Deoxy-Δ12, 14 prostaglandinj2	In vivo	Tracheal regeneration	DHAM impregnated with nanoparticles could provide support for the healing of the tracheal defect and prevent reduction of its lumen	[148]
Exosome	ADSCs-derived exosomes	In vivo	Diabetic wound healing	DAM and ADSCs-derived exosomes has more effect on diabetic wound healing	[149]
Exosome	ADSCs-derived exosomes	In vivo	Wounds	The DAM-Exos dressing accelerated wound healing by controlling inflammation, improving vascularization, and encouraging the synthesis of ECM	[124]

## Data Availability

The data that support the findings of this study are available from the corresponding author upon reasonable request.

## Nomenclature

3T3: 3-Day transfer, inoculum 3 × 10^5^ cells
15d-PGJ2: 15-Deoxy-Δ12,14-prostaglandin J2
ACL: Anterior cruciate ligament
AD-MSCs: Adipose-derived MSCs
ADSCs: Adipose-derived stem cells
AECs: Amniotic epithelial cells
ALP: Alkaline phosphatase
APCs: Apical papilla cells
ARG1: Arginase 1
BPTB: Bone-patellar tendon-bone
BRCP1: Bangladesh Regional Connectivity Project-1
CABG: Coronary artery bypass graft
CCK-8: Cell Counting Kit 8
CDH1: Cadherin-1
CSF: Cerebrospinal fluid
CYP2B6: Cytochrome P450 2B6
CYP3A4: Cytochrome P450 3A4
CYP7A1: Cytochrome P450 7A1
DAWL: Deleted in azoospermia-like
DAM: Decellularized amniotic membrane
DFUs: Diabetic foot ulcers
ECM: Extracellular matrix
ECs: Endometrial cells
EDC: 1-Ethyl-3(3-dimethyl aminopropyl)-carbodiimide
EGF: Epidermal growth factor
EMT: Epithelial-to-mesenchymal transition
EpCAM: Epithelial cellular adhesion molecule
EVs: Extracellular vesicles
GAG: Glycosaminoglycan
GP: Genipin
GVHD: Graft-versus-host disease
HAP: Human apical papilla
HLCs: Hepatocyte-like cells
HPL: Human periodontal ligament
HSV: Herpes simplex virus
IHC: Immunohistochemistry
IGF-1: Insulin-like growth factor 1
IL-10: Interleukin 10
iNOS: Inducible nitric oxide synthase
iPSCs: Induced pluripotent stem cells
K252a: Kinase inhibitor
LEC: Limbal epithelial cell
LAD: Left anterior descending
MEK1: Mitogen-activated protein kinase kinase 1
MEK2: Mitogen-activated protein kinase kinase 2
MI: Myocardial infarction
MSCs: Mesenchymal stem cells
MRC1: Mannose receptor C-type 1
MTT: (3-(4,5-Dimethylthiazol-2-yl)-2,5-diphenyltetrazolium bromide)
Muc16: Mucin 16 cell
NF: Neurofilaments
NPs: Nanoparticles
PDGF: Platelet-derived growth factor
PFMD: Pelvic floor muscle dysfunction
PFF: Pelvic fascia's fibroblast
PL-MSCs: Placenta-derived MSCs
PLGA: Poly(lactic-co-glycolic acid)
POS: Physical outstretch
PRP: Platelet-rich plasma
PUF: Penile urethrocutaneous fistulae
RM: Regenerative medicine
RT-qPCR: Reverse transcription quantitative real-time PCR
SCs: Stem cells
TCP: Tissue culture plastic
TE: Tissue engineering
TNF: Tumor necrosis factor
TPP: Tripolyphosphate
UV: Ultraviolet
VEGFs: Vascular endothelial growth factors
α-MEM: α-Minimum essential medium
